# Causal demonstration of adiposity-induced adipose-specific signaling derangements in the pathogenesis of the clinical features of the cardiovascular-kidney metabolic syndrome

**DOI:** 10.1186/s12933-026-03103-5

**Published:** 2026-02-10

**Authors:** Milton Packer

**Affiliations:** 1 Baylor Heart and Vascular Institute, Baylor University Medical Center, 621 North Hall Street, Dallas, TX USA; 2 Imperial College, London, UK

**Keywords:** Adipokines, Cardiovascular-kidney-metabolic, Heart failure

## Abstract

The American Heart Association has recognized that excess dysfunctional fat and its secretion of adipokines are upstream causal mechanisms that drive the clinical and pathophysiological features of the cardiovascular-kidney-metabolic (CKM) syndrome. The identity of these signaling molecules can be discerned by experimental adipose tissue-specific silencing studies showing that biological events induced by adiposity and occurring selectively in adipose tissue can promote each element of the CKM syndrome. Suppression of the renin-angiotensin system specifically and selectively only in adipose tissue alleviates hypertension and atherosclerosis, whereas overexpression of the mineralocorticoid receptor specifically and only in adipocytes promotes vascular injury. Silencing of autotaxin, platelet-derived growth factor D, resistin and microRNA-410-5P selectively and specifically only in adipose tissue ameliorates the development of cardiac hypertrophy and fibrosis, thus preventing experimental HFpEF. The suppression of cytoprotective adipokines (e.g., adiponectin) simultaneous with the activation of proinflammatory adipokines (e.g., leptin) promotes renal tubular sodium reabsorption and plasma volume expansion. Experimental aldosterone-induced kidney injury is accompanied by augmented expression of lipocalin-2 in visceral adipose tissue, and selective and specific deletion of lipocalin-2 only in adipose tissue—but not in the kidney—counteracts renal inflammation and fibrosis. Silencing of fatty acid binding protein 4 and 12/15-lipoxygenase selectively and specifically in adipose tissue prevents the development of insulin resistance. These observations support the conceptual framework that the accumulation of dysfunctional fat can promote each of the pathophysiological features of the CKM syndrome by altering the expression and/or secretion of signaling molecules in an adipose tissue-specific and -selective manner.

## Introduction

Visceral adiposity (identified clinically as abdominal obesity, with a waist-to-height ratio ≥ 0.5) is a defining feature of the metabolic syndrome. It is also now recognized as the earliest stage of the recent metamorphosis of the metabolic syndrome into the cardiovascular-kidney metabolic (CKM) syndrome [1]. The American Heart Association (AHA) has specifically proposed that the clinical and pathophysiological features of the CKM syndrome stem from excess visceral adipose tissue, typically related to ectopic fat depots surrounding or residing within major organs (e.g., heart, skeletal muscle, liver and kidney), which exert deleterious endocrine and paracrine actions. Inflammation in epicardial, pericardial, perivascular and perirenal adipose tissue is particularly likely to exert adverse structural and functional effects. The dysfunctional transformation of these visceral adipose tissue stores is believed to be responsible for the parallel evolution and progression of the cardiac, renal and metabolic abnormalities that (considered collectively) characterize the CKM syndrome.

How does an expansion and dysfunctional transformation of adipose tissue exert adverse effects on the cardiovascular system, the kidney and on metabolism? The AHA recognizes that visceral adiposity is accompanied by a change in the biological features and the secretory pattern of signaling molecules (adipokines) from adipose tissue [2]. In healthy lean people, adipose tissue adopts a quiescent biological profile and secretes adipokines that act to reduce cellular stress, maintain healthy homeostasis, and exert cytoprotective at distant sites in diverse organs. In contrast, in people with abdominal obesity, the expanded and inflamed visceral fat depots induce an activated state that alters signaling pathways, and consequently, they secrete adipokines that increase cellular stress and exert prohypertrophic, proinflammatory and profibrotic effects in the heart, vasculature and kidney [2]. 

This adiposity-mediated transformation in adipose tissue-specific biological events is well-positioned to produce the clinical and pathophysiological features of the CKM syndrome. Yet, we should ask: Is there direct evidence to support this hypothesis? The most compelling evidence for a causal role for adipose biological events is provided by experimental studies in which overexpression or silencing of a pathway or molecule selectively and specifically in adipose tissue can act to exacerbate or alleviate the individual clinical and pathophysiological features of the CKM syndrome.

## Visceral adiposity, adipokine imbalances and the cardiovascular system

Experimental adipose tissue-specific silencing studies show that biological events occurring specifically and selectively in adipose tissue are capable of promoting the development of the primary cardiovascular manifestations of CKM—hypertension, atherosclerosis, and heart failure with a preserved ejection fraction (HFpEF).

Mendelian randomization studies point to a causal link between visceral adiposity and hypertension [3], and both experimental and clinically, obesity is accompanied by activation of activity of the renin-angiotensin-aldosterone system. Biological events induced by adiposity and occurring specifically within adipose tissue play a critical role in the pathogenesis of this linkage, since the suppression of angiotensinogen specifically and selectively only in adipocytes alleviates the development of hypertension [4], and conversely, overexpression of the mineralocorticoid receptor specifically and only in adipocytes promotes vascular injury [5]. Activation of the renin-angiotensin system in obesity is also accompanied by upregulation of the stress-activated protein kinase, c-Jun N-terminal kinase (JNK) [6]. Suppression of JNK specifically and selectively only in adipose tissue promotes upregulation of adiponectin (an anti-inflammatory adipokine), thereby ameliorating the progression of hypertension and vascular injury [7]. 

Analogously, suppression of the transcription factor hypoxia-inducible factor-1α—specifically and selectively only in adipose tissue — can prevent the development of atherosclerosis [8]. Furthermore, transplantation of perivascular fat can promote or ameliorate atherosclerosis in a recipient mouse depending on the proatherogenic or anti-atherogenic phenotype of the donor mouse. The favorable effects of fat transplantation can be blocked by silencing of adiponectin in donor adipose tissue [9], and conversely, the proatherosclerotic actions can be inhibited by silencing of the angiotensin II receptor or the proinflammatory adipokines, monocyte chemoattractant protein-1 and angiopoietin-like protein 2, within transplanted fat [10–12]. 

Importantly, the secretion of stress-enhancing adipokines has been shown to promote hypertrophy, inflammation and fibrosis in the heart, leading to myocardial stiffening and the development of HFpEF. At the same time, proinflammatory adipokines can exert direct deleterious effects on large arteries (to cause vascular stiffness and promote systolic hypertension) and also on the coronary microvasculature (to cause coronary microvascular dysfunction and rarefaction [2]. A causal role for maladaptive adipokine signaling in the pathogenesis of HFpEF is supported by the findings from experimental studies that (1) visceral adiposity is accompanied by alteration in the expression of signaling molecules only in adipose tissue, and not in the heart or kidney; and (2) silencing of specific adipokines—e.g., autotaxin, platelet-derived growth factor D, resistin and microRNA-410-5P—selectively only in adipose tissue ameliorates the development of cardiac hypertrophy and fibrosis as well as vascular stiffness, thus preventing the development of experimental HFpEF [2]. Therefore, in experimentally-induced visceral adiposity, adipokines that are released from adipose tissue into the circulation are causally linked to the development of HFpEF.

## Visceral adiposity, adipokine imbalances and the kidney

The altered secretory profile of adipokines in people with visceral adiposity can exert two important actions on the kidney. First, the suppression of cytoprotective adipokines (e.g., adiponectin) simultaneous with the activation of proinflammatory adipokines (e.g., leptin)—acting in concert—causes enhanced renal tubular sodium reabsorption [13, 14]. The resulting sodium retention causes plasma volume expansion, thus promoting hypertension and leading to increased cardiac filling pressures, whose magnitude is directly related to body mass index in patients with HFpEF and obesity [15]. 

Second, stress-enhancing molecules secreted from adipose tissue during states of excess adiposity promote the development of glomerular and tubulointerstitial inflammation and fibrosis. Leptin promotes experimental renal injury [16], and circulating levels of leptin presage the development of chronic kidney disease in the general community [17]. Experimental aldosterone-induced kidney injury is accompanied by augmented expression of lipocalin-2 in visceral adipose tissue, and importantly, selective and specific deletion of lipocalin-2 only in adipose tissue—but not in the kidney—counteract the development of inflammation and fibrosis in the kidney [18]. 

## Visceral adiposity, adipokine imbalance and metabolic disorders

Visceral adiposity is the principal driver of insulin resistance, because biological events occurring specifically in adipose tissue are critically important regulators of whole-body insulin sensitivity. Mendelian randomization studies point to a causal relationship between visceral fat mass and type 2 diabetes [3]. Experimental down-regulation of the insulin-sensitive transporter glucose transporter 4 (GLUT4)—specifically and selectively in adipocytes—is sufficient to produce systemic insulin resistance [19]. Adiposity-mediated down-regulation of GLUT4 in adipose tissue can promote lipolysis, and the resulting increase in circulating free fatty acids promotes the hepatic overproduction of glucose, while impairing peripheral glucose utilization, i.e., the key features that characterize the development of insulin resistance in people with type 2 diabetes.

Additionally, many stress-reducing and stress-enhancing adipokines released from dysfunctional fat are known to modulate insulin sensitivity [2]. Adipose-specific secretion of the adipokine, microRNA-210-3p, suppresses GLUT4 and promotes insulin resistance [20]. Proinflammatory lipocalins—retinol binding protein 4 and fatty acid binding protein 4 (FABP4)—promote insulin resistance; suppression of FABP4 selectively and specifically in adipose tissue enhances insulin sensitivity [21, 22]. Adipose tissue-specific silencing of 12/15-lipoxygenase (12/15-LOX)—an enzyme that promotes the synthesis of bioactive lipids that act as adipokines in an endocrine manner to promote inflammation—prevents the development of insulin resistance, pancreatic islet cell injury and diabetes [23]. Of note, 12/15-lipoxygenase has also has been implicated in the pathogenesis of adverse cardiac remodeling [24]. Conversely, adipocyte-specific upregulation of sirtuin-1 and down-regulation of mammalian target of rapamycin 2 (two intracellular cytoprotective signaling molecules) promote and impair systemic insulin sensitivity, respectively [25, 26]. 

## Conclusions

**Fig. 1:**
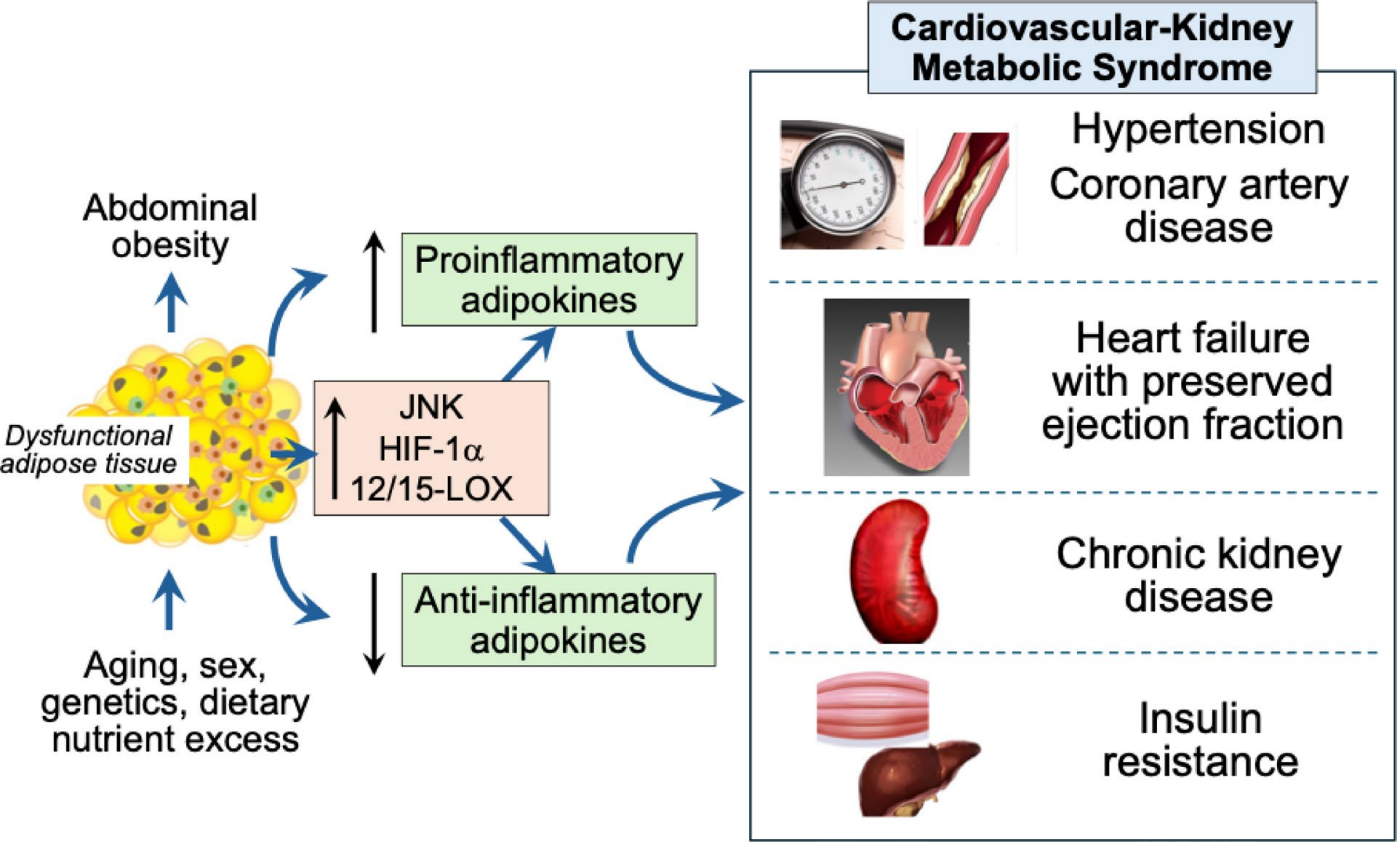
Adipose-specific biological events and secretory profiles that provide a causal link between dysfunctional fat, abdominal obesity and the evolution of the cardiovascular-kidney-metabolic syndrome. HIF-1α, hypoxia-inducible factor-1alpha; JNK, c-Jun N-terminal kinase; 12/15LOX, 12/15-lipoxygenase

Abdominal obesity and visceral adiposity are the leading diagnostic indicators and pathogenetic mechanisms, respectively, of the CKM syndrome. The observations summarized herein support the conceptual framework that dysfunctional fat can promote each of the pathophysiological features of the CKM syndrome by altering the expression and/or secretion of signaling molecules specifically and selectively only within adipose tissue, thus promoting hypertension, vascular disease and HFpEF, renal sodium retention and chronic kidney disease, and insulin resistance and dysglycemia, Fig. 1. A specific causal role for adipose tissue hypertrophy and inflammation in the CKM syndrome is highlighted by the findings of experimental studies demonstrating that molecular events specifically and selectively occurring only in adipose tissue can promote the specific key end-organ injuries that (taken together) comprise the CKM syndrome.

These observations underscore the importance of targeting visceral adiposity in preventing and treating the clinical consequences of the CKM syndrome. Glucagon-like peptide receptor-1 agonists, sodium-glucose cotransporter-2 inhibitors and mineralocorticoid receptor antagonists ameliorate visceral adiposity and act to normalize the biological derangements of dysfunctional fat [2]. Such effects likely explain their favorable effects in patients with heart failure, chronic kidney disease and insulin resistance. Novel compounds that act specifically to correct derangements in adipose tissue biology (e.g., adipokine modulators [2]) may be particularly promising in slowing the evolution and progression of the CKM syndrome. Demonstration of their efficacy in a randomized controlled clinical trial would confirm the clinical relevance of the experimental observations summarized in this paper.

## Data Availability

No datasets were generated or analysed during the current study.
